# The long-term course and relationship with survival of multidimensional fatigue in patients with brain metastases after Gamma Knife radiosurgery

**DOI:** 10.1007/s00432-023-04857-1

**Published:** 2023-05-30

**Authors:** Eline Verhaak, Wietske C. M. Schimmel, Margriet M. Sitskoorn, Patrick E. J. Hanssens, Elke Butterbrod, Karin Gehring

**Affiliations:** 1 Department of Neurosurgery–Gamma Knife Center, Elisabeth-TweeSteden Hospital, Hilvarenbeekseweg 60, 5022 GC Tilburg, The Netherlands; 2 Department of Cognitive Neuropsychology, Tilburg University, Tilburg, The Netherlands; 3 Department of Clinical, Neuro- and Developmental Psychology, Vrije Universiteit Amsterdam, Amsterdam, The Netherlands

**Keywords:** Brain metastases, Cancer, Fatigue, Multidimensional fatigue inventory, Patient reported outcomes, Radiosurgery

## Abstract

**Purpose:**

The aims of this study were to evaluate long-term multidimensional fatigue in patients with brain metastases (BM) up to 21 months after Gamma Knife radiosurgery (GKRS) and (change in) fatigue as predictor of survival.

**Methods:**

Patients with 1 to 10 BM, expected survival > 3 months, and Karnofsky Performance Status ≥ 70, and Dutch non-cancer controls were included. Fatigue was measured with the Multidimensional Fatigue Inventory. Levels of fatigue between patients and controls were compared using independent-samples t-tests. Linear mixed models were used to evaluate fatigue within the patient group up to 21 months after GKRS. Pre-GKRS fatigue and minimal clinically important (MCI) changes in fatigue in the first three months (defined as a 2-point difference) after GKRS were evaluated as predictors of survival time.

**Results:**

Prior to GKRS, patients with BM (n = 92) experienced significantly higher fatigue on all subscales than controls (n = 104). Over 21 months, physical fatigue increased, and mental fatigue decreased significantly. More specifically, general, and physical fatigue increased significantly between pre-GKRS and 3 months, followed by stable scores between 3 (n = 67) and 6 (n = 53), 6 and 12 (n = 34) and 12 and 21 (n = 21) months. An MCI increase in general or physical fatigue over the first 3 months after GKRS was a significant predictor of shorter survival time.

**Conclusion:**

Except for mental fatigue, all aspects of fatigue remained elevated or further increased up to 21 months after treatment. Furthermore, an increase in general or physical fatigue within three months after GKRS may be a prognostic indicator for poorer survival.

**ClinicalTrials.gov identifier:**

NCT02953756, November 3, 2016.

**Supplementary Information:**

The online version contains supplementary material available at 10.1007/s00432-023-04857-1.

## Introduction

Fatigue is a very distressing symptom for many patients with brain metastases (BM) (Bower and Lamkin 2013; Verhaak et al. 2019b). For example, fatigue may hamper social interactions with others and everyday tasks (Ahlberg et al. 2003; Curt et al. 2000; Magnusson et al. 1999; Verhaak et al. 2019b). Fatigue can be best assessed with a multidimensional self-report questionnaire, as fatigue is a complex symptom with physical, emotional, and mental aspects (Ahlberg et al. 2003; Jacobsen 2004; Stone and Minton 2008).

Already before treatment, patients with BM experience more fatigue as compared to the general population (Habets et al. 2016; Noh and Walbert 2018; van der Meer et al. 2018; Verhaak et al. 2019b). In our previous study on fatigue after Gamma Knife radiosurgery (GKRS) (Verhaak et al. 2019b), patients’ general and physical fatigue increased over 6 months, while mental fatigue decreased during this period. We concluded that different aspects of fatigue showed different patterns over time in patients with BM after GKRS (Verhaak et al. 2019b). Habets et al. (2016) and van der Meer et al. (2018) also reported a significant increase of fatigue in patients with BM over 6 months after stereotactic radiosurgery (SRS). These previous studies (Habets et al. 2016; van der Meer et al. 2018; Verhaak et al. 2019b) on (multidimensional) fatigue in patients with BM after SRS evaluated patients up to 6 months after SRS. Since life expectancy of patients with BM is increasing (Johnson et al. 2015; Nayak et al. 2012), insight in fatigue beyond 6 months after treatment is becoming more important.

In patients with breast cancer (Groenvold et al. 2007) and patients with high-grade glioma (Brown et al. 2006; Peters et al. 2014), fatigue has been shown to be a prognostic factor, specifically for overall survival. A possible underlying mechanism for the relationship between (increases in) fatigue and poorer survival might be progressive disease, as both fatigue and survival are related to tumor burden (Ahlberg et al. 2003; Bower and Lamkin 2013; Kurzrock 2001; Stone and Minton 2008). Over the course of their disease, patients with BM show the most change in fatigue in the initial three months after GKRS (Verhaak et al. 2019b). The association between early change in fatigue after GKRS and survival has not yet been investigated in patients with BM. Hence, it is unknown if, and to what extent, patients who show early increase in fatigue may be at risk for poorer survival. If an early increase in fatigue is indeed a prognostic indicator, early detection of increased levels of fatigue may aid early identification of patients at risk for progressive disease and/or a poorer prognosis. This may allow for these patients and their caregivers to be more closely monitored and/or counseled where needed.

The current study investigated fatigue over time in patients with BM up to 21 months after GKRS. In addition, pre-GKRS fatigue and changes in fatigue in the first three months after GKRS were evaluated as predictors of survival duration.

## Methods

The current study is a follow-up of our earlier study (Verhaak et al. 2019b) on multidimensional fatigue from pre-GKRS up to 6 months after GKRS. Data were collected as part of Cognition And Radiation-study A (CAR-Study A; ClinicalTrials.gov Identifier: NCT02953756) which was approved by the Medical Ethics Committee Brabant (file NL53472.028.15). Results regarding cognitive functioning and HRQoL in this sample have previously been described (Schimmel et al. 2020, 2021; Verhaak et al. 2019a, 2021a, 2021b).

### Patients

As previously described (Verhaak et al. 2019b), adult patients with BM, scheduled for GKRS, were recruited at the Elisabeth-TweeSteden Hospital in Tilburg, the Netherlands. Most important inclusion criteria were: 1–10 newly diagnosed BM on a contrast enhanced volumetric MRI-scan, total volume of the BM ≤ 30 cm^3^and Karnofsky performance status (KPS) ≥ 70. Most important exclusion criteria were: small cell lung cancer, a second active primary tumor and prior brain radiation or surgery.

A radiation-oncologist screened for study eligibility during the first consultation visit. Eligible patients received detailed information about the study and its procedures. In the morning before GKRS, a neuropsychological assessment, consisting of 6 short neuropsychological tests and 3 self-report questionnaires, concerning anxiety and depression (Hospital Anxiety and Depression Scale; HADS (Zigmond and Snaith 1983)), fatigue (Multidimensional Fatigue Inventory; MFI (Smets et al. 1995)), and HRQoL (Functional Assessment of Cancer Therapy-Brain; FACT-Br (FACIT.org 2017)), was scheduled for participating patients. It took approximately 60 min to complete the tests and questionnaires.

Follow-up tests and questionnaires took place every 3 months up to 21 months after GKRS and were combined with the usual care MRI-scans and consultations with the radiation-oncologist. The MRI-scans during follow-up were T1-weighted, contrast-enhanced images at 1.5 mm slice thickness. At time of treatment and at each follow-up, the total volume of the BM was determined. Partial response was defined as a ≥ 65% decrease in total tumor volume and no new BM. Progressive disease was defined as a ≥ 73% increase in total tumor volume or the appearance of new BM. Stable disease was defined as no partial response nor progressive disease. Only target lesions (lesions > 0.523 cm^3^) were used to evaluate treatment response (Lin et al. 2015).

In addition, adult Dutch non-cancer controls completed the same tests and questionnaires every 3 months up to 6 months after the first measurement (for more information refer to Verhaak et al. (2019b)). Inclusion criteria were no (history of) cancer and no cerebrovascular disease in the past 12 months. All patients and controls gave written informed consent.

### Measures

The MFI is a self-report questionnaire to measure five aspects of fatigue: general fatigue, physical fatigue, mental fatigue, reduced activity, and reduced motivation (Smets et al. 1995). The questionnaire consists of 20 items, each with a 5-point scale to indicate to what extent a given statement applies based on the preceding week (range 4 to 20 points per aspect of fatigue). Higher scores indicate more fatigue (Smets et al. 1995, 1996). Total scores for each aspect of fatigue were only calculated if all items were completed. Demographic and clinical characteristics were retrieved from patients’ medical files.

### Statistical analysis

Statistical analyses were performed with the Statistical Package for the Social Sciences (SPSS) version 24.0 (IBM Corporate Headquarters, Armonk, New York) and R (R Core Team 2017), version 3.6.1. A corrected significance level, by employing the procedure of Benjamini-Hochberg (Benjamini and Hochberg 1995), was used to correct for the false discovery rate. Descriptive statistics were used to evaluate patients’ demographic and clinical characteristics. Kaplan–Meier curves were used to analyze overall survival.

Independent-samples t-tests were conducted to investigate potential differences in mean raw MFI scores between the total group of patients with BM and Dutch controls at pre-GKRS, 6, 12, and 21 months (using controls’ first-assessment scores at each comparison). Glass’s delta effect sizes were calculated for each MFI scale. An effect size ≤ 0.49 was considered a ‘small’ effect, from 0.50 to 0.79 a ‘medium’ effect and ≥ 0.80 a ‘large’ effect (Cohen 1988). For analyses at the individual level, mean raw fatigue scores were converted into Z scores using the following formula: Z score = Y_o− _Y_p_/SD_residual_. Y_o_ is the individuals raw fatigue score, Y_p_ is the predicted raw fatigue score using regression-based formulae (based on our own control group, including age and sex as covariates), and SD_residual_ is the control group’s standard deviation (SD) of the residual. Lower Z scores indicate more severe fatigue. A Z score ≤ − 1.30 (90th percentile) was defined as ‘high fatigue’ (Bouma et al. 2012; Lezak et al. 2012). Chi-square tests for homogeneity were conducted for each aspect of fatigue to compare the proportions of patients with high fatigue with the proportion of controls with high fatigue at pre-GKRS, 6 months, 12 months, and 21 months (first-assessment scores for the controls).

The nlme package (Pinheiro et al. 2018) in R (R Core Team 2017) was used to perform linear mixed models (LMM) of the relationship of each fatigue scale with time within the group of patients with BM. The restricted maximum likelihood estimate (REML) method was used to estimate model parameters. To estimate model fit, the Akaike Information Criterion (AIC) and Bayesian Information Criterion (BIC) were used. Intercepts for the effect of fatigue were added as random intercepts to ensure that before a general trend was estimated the data over time were estimated individually for each patient. Random slopes did not improve model fit, and were therefore not added (West et al. 2014). A first-order autoregressive covariance structure (AR1) at level 1 and a Scaled Identity matrix at level 2 provided the best fit. Additionally, time was added as categorical variable to examine differences in fatigue between pre-GKRS and 3, 3 and 6, 6 and 12, and 12 and 21 months.

Although LMM can deal with missing data, there is a risk of biased results if the amount of missing data is substantial, and the data is missing in a non-random pattern. In studies regarding patients with BM, a high dropout rate is common due to short survival (Leung et al. 2011; Verhaak et al. 2020; Wong et al. 2008). To investigate whether the results of the longitudinal course of fatigue are also generalizable to the long-term survivors in our sample specifically, we also performed the LMM in the subgroup of patients who at least completed the assessment ≥ 12 months post-GKRS.

At the individual level, minimal clinically important (MCI) changes in fatigue between the above-mentioned intervals were calculated. Based on Purcell et al. (2010), an MCI increase in fatigue was defined as a 2-point increase between time-points. The number of patients with stable/decreased or increased fatigue were counted.

The survival Package (Therneau 2021) in R (R Core Team 2017) was used to create Accelerated Failure Time (AFT) models to evaluate the 5 subscales of fatigue as predictors of survival time (time between GKRS and date of death). The AFT model can fit to lognormal distribution of the survival data in our sample and computes a Time Ratio (TR) that expresses the effect of a predictor into an increase (TR > 1) or decrease (TR < 1) in survival duration. First, we adopted the clinical prognostic factors age, KPS (0 = 90–100, 1 = 70–80), volume of BM (0 = medium (between 4.8 and 12.6 cm^3^), 1 = small (< 4.8 cm^3^), 2 = large (> 12.6 cm^3^)(Habets et al. 2016)), histology (0 = other, 1 = non-small cell lung cancer (NSCLC)), extracranial metastases (0 = no, 1 = yes), and number of BM (triple-dose contrast enhanced MRI)(Achrol et al. 2019; Lu-Emerson and Eichler 2012; Nieder et al. 2000; Sperduto et al. 2012) to a clinical base model (model 1). Significant predictors of survival (*p* < 0.05) were kept in this model.

In a second model (model 2), the 5 continuous pre-GKRS raw fatigue subscale scores were separately added as predictor (5 different models in total) to the clinical base model. Potential sociodemographic covariates of fatigue were evaluated with Pearson correlation (age), point-biserial correlation (sex), and Spearman’s correlation (educational level). In case of a significant (*p* < 0.05) correlation, the significant covariate was added to model 2 of the relevant fatigue subscale.

In a third model (model 3), an MCI increase in fatigue between pre-GKRS and 3 months thereafter (0 = stable/declined fatigue versus 1 = increased fatigue) was added as a separate predictor to the clinical base model (5 different models in total), as our previous study (Verhaak et al. 2019b) showed the most change in fatigue during this interval. Potential sociodemographic covariates of an MCI increase in fatigue were evaluated with point-biserial correlation (age), Fisher exact (sex), and Chi-square test of independence (educational level). In case of a significant (*p* < 0.05) correlation, the significant covariate was added to model 3 of the relevant fatigue subscale. In case an MCI increase in a fatigue subscale was a significant predictor, estimations of overall survival of patients with similar clinical characteristics, but stable/declined versus increased fatigue were computed with a multivariate estimation of median time to event.

## Results

### Characteristics and compliance

**Fig. 1:**
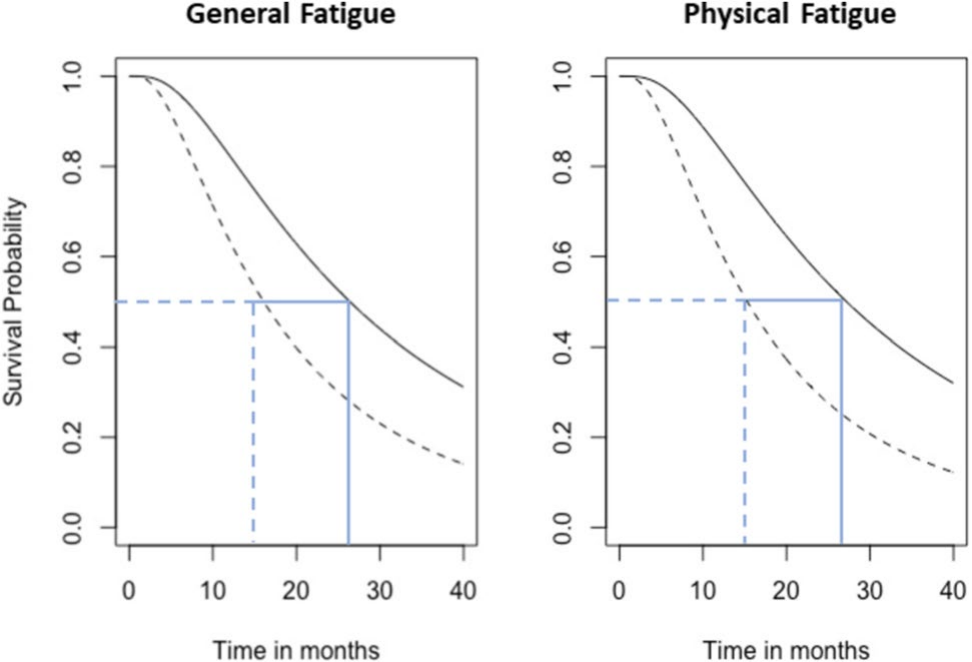
Multivariate survival probabilities of general and physical fatigue over time. Solid line: patients with Karnofsky performance status (KPS) 90–100, primary non-small cell lung cancer (NSCLC), and stable general fatigue or physical fatigue. Dashed line: patients with KPS 90–100, primary NSCLC, and a minimal clinically important (MCI) increase in general fatigue or physical fatigue

Baseline characteristics of the 92 patients (See Supplementary data for the selection process) with BM and 104 Dutch controls were comparable regarding age, sex, and education (Table 1). The median overall survival was 11.8 months (95% CI 8.6 to 15.0 months); 18 patients (19.6%) were censored. The one-year survival rate was 48.9%. The median survival of patients with a follow-up at or beyond 12 months (n = 38) was 39.9 months (95% CI 18.3 to 61.5 months); 16 patients (42.1%) were censored. Of the 67 patients with at least one follow-up assessment, 41 patients (61.2%) had intracranial progression (solely due to new lesions in 19 patients (46.3%)), 11 patients (16.4%) had a partial response, and 15 patients (22.4%) had stable disease. Reasons for dropout were death (n = 33), assessment too burdensome (n = 19), no clinical follow-up due to poor neurological or physical condition (n = 16), clinical follow-up in a different hospital (n = 2), and not able to complete the MFI (n = 1). The reasons for dropout for the long-term survivors were previously published in Verhaak et al. (2021b); Fig. 1 Patient flowchart.

**Table 1:** Patient characteristics

Baseline characteristics	No. of patients (%)	Control group (%)
Number of patients	92 (100)	104 (100)
Age in years, median (range)	63.0 (31–80)	60 (31–87)
Sex		
Male	47 (51.1)	50 (48.1)
Female	45 (48.9)	54 (51.9)
Education level^a^		
Low	28 (30.4)	25 (24.0)
Middle	37 (40.2)	33 (31.7)
High	27 (29.3)	46 (44.2)
No. of brain metastases^b^		
1	32 (34.8)	
2–4	29 (31.5)	
5–10	31 (33.7)	
Diagnosis of BM^c^		
Synchronous	28 (30.4)	
Metachronous	64 (69.6)	
KPS, median (range)	90 (70–100)	
70–80	33 (35.9)	
90–100	59 (64.1)	
RPA		
Class 1	16 (17.4)	
Class 2	76 (82.6)	
GPA		
Class 2	15 (16.3)	
Class 3	60 (65.2)	
Class 4	17 (18.5)	
Primary tumor site		
Lung (NSCLC)	55 (59.8)	
Renal	15 (16.3)	
Melanoma	12 (13.0)	
Breast	6 (6.5)	
Other	4 (4.4)	
Systemic treatment before or at GKRS		
No	39 (42.4)	
Yes	53 (57.6)	
Chemotherapy	17 (18.5)	
Chemo-radiotherapy	11 (12.0)	
Targeted therapy	11 (12.0)	
Chemo- and immunotherapy	4 (4.3)	
Chemo- and targeted therapy	3 (3.3)	
Chemo- and hormonal therapy	2 (2.2)	
Immuno- and targeted therapy	2 (2.2)	
Immunotherapy	1 (1.1)	
Chemo-, immuno-, and hormonal therapy	1 (1.1)	
Chemo-, immuno-, hormonal, and targeted therapy	1 (1.1)	
Use of dexamethasone at GKRS		
No	29 (31.5)	
Yes	63 (68.5)	
Total BM volume cm^3^, median (range)^d^	5.6 (0.02–31.15)	
Small (< 4.8 cm^3^)	40 (43.5)	
Medium (4.8–12.6 cm^3^)	25 (27.2)	
Large (> 12.6 cm^3^)	27 (29.3)	

*No*. number, *mo* months, *KPS* Karnofsky performance status, *RPA* recursive partitioning analysis, *GPA* graded prognostic assessment, *NSCLC* non-small cell lung cancer, *BM* brain metastases

^a^The 7 categories to classify the level of education of the Verhage scale (Verhage 1964) were merged into low (Verhage 1–4), middle (Verhage 5), and high (Verhage 6 and 7) educational level

^b^On the MRI-scan used for treatment planning

^c^Diagnosis of BM within (synchronous) or after 30 days (metachronous) of the diagnosis of the primary tumor

^d^One patient had a total tumor volume 31.15 cm^3^ on the MRI-scan used for treatment planning

### Fatigue status

Mean raw fatigue scores of the patients at each time-point are presented in Supplemental Table S1. Patients with BM experienced significantly higher levels of fatigue on all subscales at pre-GKRS and at 3, 6, and 12 months after GKRS compared to controls (*p* ≤ 0.007; Table 2). At 21 months, patients experienced significantly higher levels of fatigue for general and physical fatigue, but not for mental fatigue, reduced activity, and reduced motivation. Largest effect sizes (1.0–1.2) for differences between patients and controls were found for reduced activity (pre-GKRS, 3, 6, and 12 months), general fatigue (3, 6, and 12 months), and physical fatigue (3, 6, and 12 months).

**Table 2:** Fatigue scores (MFI) of patients with BM and controls

	Control group (n = 102^¥^) mean (SD)	Patients with BM mean raw fatigue scores (SD)					Patients with BM versus the control group														
							T0			T3			T6			T12			T21		
		T0 (n = 92)	T3 (n = 67)	T6 (n = 53)	T12 (n = 34)	T21 (n = 21)	t	p*	ES	t	p*	ES	t	p*	ES	t	p*	ES	t	p*	ES
General fatigue	8.8 (3.8)^a^	11.5 (4.3)	13.2 (4.5)	13.1 (4.6)	12.7 (4.6)	11.6 (4.4)	4.7	** < 0.001**	0.7	6.8	** < 0.001**	1.2	6.2	** < 0.001**	1.1	4.9	** < 0.001**	1.0	3.0	**0.004**	0.7
Physical fatigue	8.6 (4.2)	10.7 (4.6)	13.1 (4.8)	13.2 (4.9)	12.7 (5.2)	11.8 (4.9)	3.4	**0.001**	0.5	6.4	** < 0.001**	1.1	6.1	** < 0.001**	1.1	4.7	** < 0.001**	1.0	3.1	**0.002**	0.8
Mental fatigue	8.2 (3.7)	11.3 (4.0)^b^	10.4 (4.5)	10.3 (4.3)	10.3 (4.2)	9.1 (3.7)	5.5	** < 0.001**	0.8	3.4	**0.001**	0.6	3.2	**0.002**	0.6	2.7	**0.007**	0.6	1.0	0.332	0.2
Reduced activity	8.3 (3.4)^a^	11.7 (4.0)	12.4 (4.6)	11.9 (4.2)	11.7 (4.9)	10.7 (5.2)	6.5	** < 0.001**	1.0	6.3^c^	** < 0.001**	1.2	5.9	** < 0.001**	1.1	3.8^c^	** < 0.001**	1.0	2.1^c^	0.050	0.7
Reduced motivation	7.4 (3.1)	9.3 (3.8)^b^	10.1 (4.0)	10.0 (3.6)	9.7 (3.8)	8.7 (4.1)	3.7^c^	** < 0.001**	0.6	4.8	** < 0.001**	0.9	4.6	** < 0.001**	0.8	3.5	**0.001**	0.7	1.6	0.107	0.4

Higher scores indicate more fatigue. Bold text indicates statistical significance

*ES* Glass delta effect size, *MFI* multidimensional fatigue inventory, *BM* brain metastases, *n* number of participants, *SD* standard deviation, *mean diff* mean difference, *mo* months, *T0* pre-GKRS, *T3, T6, T12 and T21* 3, 6, 12, and 21 months after GKRS respectively

^*^Corrected alpha of 0.050 for pre-GKRS, 6 months, and 12 months, and 0.020 for 21 months, using the Benjamini–Hochberg procedure (Benjamini and Hochberg 1995)

^a^number of controls = 101

^b^Number of patients with BM = 91

^c^Equal variances not assumed

^¥^102/104 (98%) controls completed the MFI

At the individual level, significantly higher proportions of patients (28.3–55.9%) had high fatigue scores compared with the controls (11.8–15.7%) for all subscales at pre-GKRS, 6 months, and 12 months (*p* ≤ 0.014). At 21 months, significantly higher proportions of patients versus controls experienced reduced activity (38.1% versus 11.9%), while no significant differences were found for the other fatigue scales (Supplemental Table S2).

### Changes in fatigue

From pre-GKRS to 21 months, patients’ physical fatigue increased, and mental fatigue decreased significantly. There were no significant changes in levels of general fatigue, reduced activity, and reduced motivation over 21 months. Regarding the separate intervals, there was a significant increase in levels of general and physical fatigue between pre-GKRS and 3 months, followed by stable scores (Table 3). For the long-term survivors specifically (n = 38), there was a significant decrease in mental fatigue from pre-GKRS to 21 months. Regarding the separate intervals, there was a significant increase in physical fatigue between pre-GKRS and 3 months, followed by stable scores (Table S3).

**Table 3:** Linear mixed model results of fatigue over time of patients with brain metastases after Gamma Knife radiosurgery

	Time Slope T0-T21 beta (SE)	F-value	p^*^	Interval			
				T0–T3 *b* (SE)*	T3–T6 *b* (SE)*	T6–T12* b* (SE)*	T12–T21* b* (SE)*
General fatigue	0.15 (0.1)	2.081	0.150	**1.7 (0.5)**	− 0.3 (0.6)	− 0.0 (0.7)	− 0.5 (1.0)
Physical fatigue	**0.46 (0.1)**	**10.860**	**0.001**	**2.3 (0.5)**	− 0.1 (0.5)	0.2 (0.7)	0.6 (0.9)
Mental fatigue	− **0.25 (0.1)**	**8.591**	**0.004**	− 0.7 (0.4)	− 0.4 (0.5)	− 0.1 (0.6)	− 0.6 (0.8)
Reduced activity	− 0.07 (0.1)	0.508	0.477	0.6 (0.5)	− 0.7 (0.5)	0.2 (0.7)	− 0.2 (0.9)
Reduced motivation	0.05 (0.1)	0.319	0.573	1.0 (0.4)	− 0.3 (0.5)	0.1 (0.6)	− 0.4 (0.8)

Bold text indicates statistical significance

*SE* standard error, *T0* pre-GKRS, *T6* 6 months, *T12* 12 months, *T21* 21 months

*Corrected alpha’s, using the Benjamini–Hochberg procedure (Benjamini and Hochberg 1995), were 0.020 for the overall models of fatigue (time slope T0-T21) and 0.013 for the separate time intervals

At the individual level as well, most patients had an MCI increase in physical fatigue (52.2%) between pre-GKRS and 3 months follow-up. For the other fatigue scales and intervals, most patients showed stable or decreased fatigue scores (Supplemental Table S4).

### Fatigue as predictor of survival

In the AFT clinical base model, age, total BM volume, number of BM, and extracranial metastases were not significantly related to survival time (*p* > 0.05), while KPS 70–80 (*p* = 0.04) and NSCLC (*p* = 0.04) were significant predictors of shorter survival time (data not shown; time ratio (TR) 0.56 and 0.54, respectively). KPS and NSCLC were kept in the clinical base model (Table 4).

**Table 4:** Prediction models of survival time in patients with BM

	Coefficient	SE	TR	p*
**Clinical base model (model 1)**				
KPS (70–80)	− 0.58	0.29	0.56	0.047
Histology (NSCLC)	− 0.52	0.29	0.59	0.073
**Pre-GKRS fatigue (model 2)**				
General fatigue				
KPS (70–80)	− 0.67	0.32	0.51	0.035
Histology (NSCLC)	− 0.55	0.29	0.58	0.060
General fatigue	0.03	0.04	1.03	0.476
Physical fatigue				
KPS (70–80)	− 0.75	0.32	0.47	0.020
Histology (NSCLC)	− 0.57	0.29	0.56	0.049
Physical fatigue	0.04	0.03	1.04	0.227
Mental fatigue				
KPS (70–80)	− 0.52	0.30	0.59	0.081
Histology (NSCLC)	− 0.46	0.29	0.63	0.112
Mental fatigue	0.01	0.04	1.01	0.754
Reduced activity				
KPS (70–80)	− 0.79	0.31	0.45	**0.012**
Histology (NSCLC)	− 0.56	0.29	0.57	0.051
Reduced activity	0.06	0.04	1.06	0.095
Reduced motivation				
Age	− 0.02	0.02	0.98	0.298
KPS (70–80)	− 0.64	0.32	0.52	0.043
Histology (NSCLC)	− 0.52	0.29	0.59	0.073
Reduced motivation	0.03	0.04	1.03	0.448
**Fatigue MCI change (model 3)**				
General fatigue				
KPS (70–80)	− 0.49	0.23	0.61	**0.033**
Histology (NSCLC)	− 0.35	0.22	0.71	0.120
MCI change in general fatigue	− 0.50	0.22	0.61	**0.023**
Physical fatigue				
KPS (70–80)	− 0.53	0.23	0.59	**0.019**
Histology (NSCLC)	− 0.42	0.22	0.65	0.054
MCI change in physical fatigue	− 0.57	0.22	0.56	**0.008**
Mental fatigue				
KPS (70–80)	− 0.44	0.24	0.65	0.065
Histology (NSCLC)	− 0.34	0.23	0.71	0.140
MCI change in mental fatigue	0.21	0.25	1.23	0.408
Reduced activity				
KPS (70–80)	− 0.47	0.23	0.63	0.043
Histology (NSCLC)	− 0.40	0.23	0.67	0.077
MCI change in reduced activity	− 0.45	0.22	0.64	0.047
Reduced motivation				
KPS (70–80)	− 0.43	0.23	0.65	0.062
Histology (NSCLC)	− 0.37	0.23	0.69	0.100
MCI change in reduced motivation	− 0.46	0.22	0.63	0.038

*BM* Brain metastases, *SE* standard error, *TR* time ratio, *KPS* Karnofsky performance score, *NSCLC *non-small cell lung cancer*, MCI* minimal clinically important

*Corrected alpha’s, using the Benjamini–Hochberg procedure (Benjamini and Hochberg 1995), were 0.025 for model 1, 0.017 (general, physical, mental fatigue and reduced motivation) and 0.013 (reduced motivation) for model 2, and 0.033 (general and physical fatigue) and 0.017 (mental fatigue, reduced activity, and reduced motivation) for model 3. Bold text indicates statistical significance

In the second model (model 2), age was added as covariate for reduced motivation (Supplemental Table S5). In model 2, none of the pre-GKRS fatigue subscale scores predicted survival time (*p* > 0.05; Table 4).

In the third model (model 3), no additional covariates were adopted (Supplemental Table S4). Early MCI increases from pre-GKRS to 3 months thereafter in general fatigue (*p* = 0.023, TR = 0.61) and in physical fatigue (*p* = 0.008, TR = 0.56) were negative predictors of survival time (i.e., an MCI increase in fatigue predicted a reduction of survival time of 39% and 44% respectively compared to stability or decreased fatigue). An MCI increase in the other fatigue scales did not significantly predict survival time (Table 4).

To illustrate, survival times for patients with similar clinical characteristics—KPS 90–100, primary NSCLC—and a stable versus an MCI increase fatigue within the first 3 months after GKRS were estimated. Estimated survival times were 26.4 months (CI 17.9–38.8 months) for stable general fatigue versus 16.1 months (CI 11.1–23.2 months) for an MCI increase in general fatigue, and 27.2 months (CI 18.7–39.6 months) for stable physical fatigue versus 15.3 months (CI 10.6–22.0 months) for an MCI increase in physical fatigue (Fig. 1).

## Discussion

At pre-GKRS, and 6 and 12 months after treatment, patients experienced significantly higher fatigue, on all aspects, compared to Dutch controls. At 21 months, patients only experienced significantly higher general and physical fatigue compared to Dutch controls. This may be explained by the more favorable characteristics of the long-term survivors, or due to statistical power issues (as the power at 21 months ranged between 0.09 and 0.87 for the five fatigue subscales).

Over the span of 21 months, physical fatigue increased, whereas mental fatigue decreased significantly, while the other aspects of fatigue did not change. For the intervals, there was a significant increase between pre-GKRS and 3 months in general and physical fatigue followed by stable scores. For the long-term survivors, a significant decrease in mental fatigue was found over the span of 21 months while none of the domains significantly increased. The long-term survivors also showed a significant increase in physical fatigue between pre-GKRS and 3 months specifically, which remained stable thereafter.

At the individual level, most patients (52.2%) had an MCI increase in physical fatigue between pre-GKRS and 3-months, followed by stable/decreased scores (58.8%–69.8% across intervals). For the other fatigue scales, most patients had stable/decreased fatigue scores across intervals (50.7%–79.2%). However, still 20.8%–49.3% of the patients experienced an MCI increase in fatigue over the different intervals.

These group level results are very similar to the results in our prior study (Verhaak et al. 2019b), regarding fatigue up to 6 months after GKRS. We argued that the increase in general and physical fatigue in the first 3 months after GKRS could be an early side effect of radiation (Jereczek-Fossa et al. 2002; Verhaak et al. 2019b). In addition, inflammatory processes may play a role in the increase in general and physical fatigue (see below for more information). Regarding the course of mental fatigue, being diagnosed with a life-threatening disease and the upcoming treatment with GKRS may lead to high mental fatigue in the period before treatment, as observed in our data. After treatment, our results demonstrate a significant decline in mental fatigue, potentially caused by a gradual decline of the mental distractions. Another explanation may be that the response shift phenomenon, in which internal standards, values, and conceptualization of self-reported health outcomes are re-assessed over time (Dirven et al. 2013; Lin et al. 2013a; Schwartz et al. 2006; Sprangers 2002; Wilson 1999), primarily affects ratings of mental fatigue, reduced activity and motivation.

An MCI increase in general or physical fatigue in the first three months after GKRS, but not pre-GKRS fatigue, predicted significant reductions in survival time, independently of significant clinical prognostic characteristics. Patients with an MCI increase in general fatigue and physical fatigue had a 39% and 44% decrease in overall survival time respectively, as compared to patients with stable/decreased fatigue scores. Considering similar KPS, similar primary tumor histology, and early increased (versus stable/declined) fatigue, absolute differences in median survival times were 10 to 12 months. This is substantial considering the overall poor prognosis for patients with BM.

In this study we included baseline predictors of survival in addition to fatigue. However, we emphasize that fatigue might be best viewed as a prognostic indicator, based on patients’ subjective experience, as opposed to an independent risk factor. There may be other factors, such as extent of disease or systemic treatment, that might influence the relation between an MCI increase in fatigue and survival. For example, tumor burden and/or progression can induce inflammatory processes (including changes in cytokine levels) and dysregulation of the hypothalamic–pituitary–adrenal axis (Ahlberg et al. 2003; Bower and Lamkin 2013; Kurzrock 2001; Stone and Minton 2008), which in turn can lead to an increased fatigue (Ahlberg et al. 2003; Bower and Lamkin 2013; Kurzrock 2001; Shi et al. 2018; Spiegel and Giese-Davis 2003; Stone and Minton 2008; Strain and Blumenfield 2018). Especially with inflammatory processes, higher physical fatigue is expected rather than mental or motivational fatigue (Karshikoff et al. 2017). The questions regarding physical and general fatigue might better reflect these inflammatory processes than the questions regarding mental and motivational fatigue. This may explain our finding that only an MCI increase in general and physical fatigue were significant predictors of survival time. An increase in the subjective experience of physical and general fatigue could therefore be a signal for unstable intra- and/or extracranial disease, especially when the increase in fatigue cannot be explained by other factors (such as a side-effect of anti-cancer treatment, mood, anxiety, personal life events, or recent activities).

Missing data are a common challenge in studies using longitudinal patient-reported outcomes, especially when survival is short and the pattern of missing data is likely related to disease status (Leung et al. 2011; Verhaak et al. 2020; Wong et al. 2008). Information on levels of fatigue of long-term survivors after stereotactic radiosurgery is unsurprisingly lacking. Nevertheless, there is a substantial group of patients who do live longer than 6–12 months after treatment. Understanding the course of one of the most common and burdensome symptoms is very relevant for these patients. Our analyses showed that the longitudinal fatigue trajectories of the entire sample and the long-term survivors were very similar, as mental fatigue decreased over time and physical fatigue stabilized after an early increase in both groups. However, our findings also indicated that general fatigue and possibly also physical fatigue show a more stable course over time in the long-term survivors than in the entire group. Notably, the survival analyses also identified these two domains as predictors of survival.

A limitation of this study is that our patient group might have been less fatigued and in better clinical condition than the general group of patients with BM selected for SRS, as they were willing to participate in a time demanding study. Second, patients may have experienced additional fatigue due to stress at the day of completion of the questionnaire as compared to daily life that may impact their ratings. However, most increase in fatigue was reported in the physical domain, while mental fatigue and motivation domains may be more susceptible to stress.

Further research is needed to disentangle the relationship between fatigue and intra- and/or extracranial disease status and survival in patients with BM. However, assessment of extracranial disease status is complex due to different follow-up protocols (diagnostic tools and scan intervals) across hospitals (Nieder and Mehta 2009). In future trials, effort should go towards aligning the time points of assessment of fatigue and intra- and extracranial disease. Furthermore, clinicians should be aware that an increase in fatigue may be an early indicator for unstable disease even before radiology scans may indicate progression. If measuring fatigue with a multidimensional tool in the clinical practice is too time consuming, clinicians may routinely ask patients about (general and physical) fatigue.

Up to 21 months after GKRS, feelings of fatigue were present for all different fatigue aspects for a substantial part of the patients, indicating that fatigue is a serious and persistent symptom in these patients. Furthermore, an increase in general or physical fatigue in the first 3 months after GRKS was a significant predictor of shorter survival. There should be more awareness in clinical practice of increased fatigue as potential warning signal for survival.

## Supplementary Information

Below is the link to the electronic supplementary material.

Supplementary file1 (DOCX 35 KB)

## Data Availability

The datasets generated during and/or analyzed during the current study are available from the corresponding author on reasonable request.
