# Supplementary material for: The long-term course and relationship with survival of multidimensional fatigue in patients with brain metastases after Gamma Knife radiosurgery
Source: J Cancer Res Clin Oncol. 2023 May 30;149(12):9891–901. doi: 10.1007/s00432-023-04857-1 (PMC10423137; doi:10.1007/s00432-023-04857-1)
Supplement: Supplementary file 1 — Supplementary file1 (DOCX 35 KB) [file 432_2023_4857_MOESM1_ESM.docx]

**Supplementary data**

**The long-term course and relationship with survival of multidimensional fatigue in patients with brain metastases after Gamma Knife radiosurgery**

**Eline Verhaak^1,2^, Wietske C.M. Schimmel^1,2^, Margriet M. Sitskoorn^1,2^, Patrick E. J. Hanssens^1^, Elke Butterbrod^1,3^, AND Karin Gehring^1,2^**

^1^ Department of Neurosurgery - Gamma Knife Center, Elisabeth-TweeSteden Hospital, Tilburg, The Netherlands

^2^ Department of Cognitive Neuropsychology, Tilburg University, Tilburg, The Netherlands

^3^ Department of Clinical, Neuro- and Developmental Psychology, Vrije Universiteit Amsterdam, Amsterdam, The Netherlands

Selection process study participation

In total, 391 patients with BM were evaluated for study participation of which 146 patients were selected as eligible, 46 patients declined participation (24 patients found participation too burdensome and 22 patients did not want to participate for other reasons). Of the 100 included patients, 8 patients were excluded from the analyses due to no BM (histologically proven 9 months after inclusion), volume of 44.8cc at day of treatment, >10 BM at day of treatment (2 patients), large hematoma, aphasia, KPS < 70 at day of treatment, diagnosis of dementia.

| **Table S1.** Fatigue scores (MFI) of patients with BM and controls | | | | | | | | |
| --- | --- | --- | --- | --- | --- | --- | --- | --- |
|  | Control Group  (n=102^¥^)  Mean raw  fatigue scores (SD) | Patients with BM  Mean raw fatigue scores (SD) | | | | | | |
|  |  | T0  (n=92) | T3  (n=67) | T6  (n=53) | T9  (n=41) | T12  (n=34) | T15  (n=28) | T21  (n=21) |
| General Fatigue | 8.8 (3.8)^a^ | 11.5 (4.3) | 13.2 (4.5) | 13.1 (4.6) | 12.9 (4.6) | 12.7 (4.6) | 12.1 (4.8) | 11.6 (4.4) |
| Physical Fatigue | 8.6 (4.2) | 10.7 (4.6) | 13.1 (4.8) | 13.2 (4.9) | 12.3 (5.3) | 12.7 (5.2) | 11.7 (4.6) | 11.8 (4.9) |
| Mental Fatigue | 8.2 (3.7) | 11.3 (4.0)^b^ | 10.4 (4.5) | 10.3 (4.3) | 9.7 (3.9) | 10.3 (4.2) | 9.9 (4.8) | 9.1 (3.7) |
| Reduced Activity | 8.3 (3.4)^a^ | 11.7 (4.0) | 12.4 (4.6) | 11.9 (4.2) | 11.7 (5.0) | 11.7 (4.9) | 10.4 (4.0) | 10.7 (5.2) |
| Reduced Motivation | 7.4 (3.1) | 9.3 (3.8)^b^ | 10.1 (4.0) | 10.0 (3.6) | 9.5 (4.1) | 9.7 (3.8) | 8.6 (2.8) | 8.7 (4.1) |
| *Note:* MFI: Multidimensional Fatigue Inventory, BM: brain metastases, n: number of participants, SD: standard deviation, T0: pre-GKRS, T3, T6, T9, T12, T15, and T21: 3, 6, 9, 12, 15, and 21 months after GKRS, respectively.  ^a^ Number of controls = 101, ^b^ Number of patients with BM = 91, ^c^ Equal variances not assumed. ^¥^ 102/104 (98%) controls completed the MFI. | | | | | | | | |

| Table S2. Percentages of high fatigue for patients with brain metastases versus controls | | | | | | | | | |
| --- | --- | --- | --- | --- | --- | --- | --- | --- | --- |
|  | **Controls** | **Patients** | | | | | | | |
|  | T0  (n=102) | T0  (n=92) | | T6  (n = 53) | | T12  (n=34) | | T21  (n = 21) | |
|  | Fatigued | Fatigued | *p*^*^ | Fatigued | *p*^*^ | Fatigued | *p*^*^ | Fatigued | *p*^*^ |
| General Fatigue | 14.9%^a^ | 32.6% | **.004** | 47.2% | **<.001** | 47.1% | **<.001** | 28.6% | .199^c^ |
| Physical Fatigue | 12.7% | 30.4% | **.003** | 50.9% | **<.001** | 55.9% | **<.001** | 33.3% | .045^c^ |
| Mental Fatigue | 11.8% | 34.1%^b^ | **<.001** | 28.3% | **.010** | 32.4% | **.006** | 9.5% | 1.000^c^ |
| Reduced Activity | 11.9%^a^ | 45.7% | **<.001** | 41.5% | **<.001** | 50.0% | **<.001** | 38.1% | **.007**^c^ |
| Reduced Motivation | 15.7% | 36.3%^b^ | **.001** | 39.6% | **.001** | 35.3% | **.014** | 14.3% | 1.000^c^ |
| Note: T0: pre-GKRS, T6: 6 months, T12: 12 months, T21: 21 months after GKRS, respectively.  ^*^A corrected alpha of .050 (pre-GKRS, 6 and 12 months) and .010 (21 months) was used (Benjamini and Hochberg 1995). Fatigued was defined as a z score ≤ -1.3. ^a^ n = 101, ^b^ n = 91, ^c^ Fisher’s exact test.  Bold type indicates statistical significance. | | | | | | | | | |

| **Table S3.** Linear mixed model results of fatigue over time in a subgroup of long-term survivors with brain metastases after Gamma Knife radiosurgery | | | | | | | |
| --- | --- | --- | --- | --- | --- | --- | --- |
|  | Time Slope T0-T21  beta (SE) | F-value | *p*^*^ | Interval | | | |
|  |  |  |  | T0-T3  *b* (SE)^*^ | T3-T6  *b* (SE)^*^ | T6-T12  *b* (SE)^*^ | T12-T21  *b* (SE)^*^ |
| General Fatigue | 0.10 (0.1) | 0.748 | .388 | 0.9 (0.7) | 0.1 (0.7) | 0.1 (0.8) | -0.5 (1.0) |
| Physical Fatigue | 0.29 (0.1) | 4.309 | .039 | **1.6 (0.6)** | 0.3 (0.6) | 0.1 (0.7) | 0.5 (0.9) |
| Mental Fatigue | **-0.26 (0.1)** | **7.817** | **.006** | -0.9 (0.6) | -0.7 (0.6) | 0.1 (0.7) | -0.6 (0.8) |
| Reduced Activity | -0.11 (0.1) | 1.216 | .272 | -0.2 (0.6) | -0.7 (0.6) | 0.4 (0.7) | -0.1 (0.9) |
| Reduced Motivation | 0.01 (0.1) | 0.005 | .941 | 0.2 (0.6) | -0.2 (0.6) | 0.3 (0.7) | -0.3 (0.8) |
| *Note:* SE: standard error, T0: pre-GKRS, T6: 6 months, T12: 12 months, T21: 21 months.  ^*^ Corrected alpha’s, using the Benjamini-Hochberg procedure (Benjamini and Hochberg 1995), were .0100 for the overall models of fatigue (time slope T0-T21) and .0125 for the separate time intervals.  Bold text indicates statistical significance. | | | | | | | |

| **Table S4.** Percentages of patients with a minimal clinically increase in fatigue | | | | | | | | |
| --- | --- | --- | --- | --- | --- | --- | --- | --- |
|  | T0-T3  (n=67) | | T3-T6  (n=53) | | T6-T12  (n=34) | | T12-T21  (n=17) | |
|  | Stable | Increase | Stable | Increase | Stable | Increase | Stable | Increase |
| General Fatigue | 50.7% | 49.3% | 77.4% | 22.6% | 61.8% | 38.2% | 70.6% | 29.4% |
| Physical Fatigue | 47.8% | 52.2% | 69.8% | 30.2% | 67.6% | 32.4% | 58.8% | 41.2% |
| Mental Fatigue | 70.1% | 29.9% | 73.6% | 26.4% | 76.5% | 23.5% | 58.8% | 41.2% |
| Reduced Activity | 58.2% | 41.8% | 79.2% | 20.8% | 58.8% | 41.2% | 64.7% | 35.3% |
| Reduced Motivation^a^ | 57.6% | 42.4% | 75.5% | 24.5% | 52.9% | 47.1% | 70.6% | 29.4% |
| Note: ^a^ n = 66 for T0-T3, T0: pre-GKRS, T3: 3 months, T6: 6 months, T12: 12 months, T21: 21 months after GKRS, respectively. | | | | | | | | |

| **Table S5.** Correlations between fatigue and age, sex, and educational level | | | | | | |
| --- | --- | --- | --- | --- | --- | --- |
|  | | General Fatigue | Physical Fatigue | Mental Fatigue | Reduced Activity | Reduced Motivation |
| *Baseline fatigue (model 2)* |  |  |  |  |  |  |
| Age | *b*  *p* value^*^ | -0.025  .810 | 0.126  .230 | -0.024  .824 | 0.195  .063 | 0.211  **.045** |
| Sex | *b*  *p* value^*^ | 0.150  .154 | 0.126  .231 | 0.151  .154 | 0.017  .869 | 0.103  .330 |
| Educational level | *b*  *p* value^*^ | 0.101  .339 | 0.079  .452 | -0.111  .295 | -0.096  .361 | -0.099  .351 |
| *Fatigue MCI difference (model 3)* |  |  |  |  |  |  |
| Age | *b*  *p* value^*^ | 0.078  .531 | -0.133  .281 | -0.014  .910 | 0.019  .878 | 0.048  .700 |
| Sex | *b*  *p* value^*^ | 0.128  .720 | 0.156  .693 | 0.018  .892 | 0.225  .635 | 0.132  .716 |
| Educational level | *b*  *p* value^*^ | 1.559  .459 | 1.309  .520 | 0.370  .831 | 1.680  .432 | 1.093  .579 |
| *MCI:* minimal clinically important. Bold text indicates statistical significance. | | | | | | |

**References**

Benjamini Y, Hochberg Y (1995) Controlling the false discovery rate: a practical and powerful approach to multiple testing Journal of the Royal Statistical Society Series B (Methodological):289-300
